# Decoding ADGRE5: How Proteolytic Cleavage and Mechanical Forces Unleash Cellular Signals

**DOI:** 10.3390/cells14161284

**Published:** 2025-08-19

**Authors:** Ana L. Moreno-Salinas, Arturo Mancini, Samya Aouad, Herthana Kandasamy, Sandra Morissette, Arhamatoulaye Maiga, Michel Bouvier, Richard Leduc, Laurent Sabbagh

**Affiliations:** 1 Department of Pharmacology-Physiology, Université de Sherbrooke, Sherbrooke, QC J1H 5N4, Canada; ana.lilia.moreno.salinas2@usherbrooke.ca; 2 Domain Therapeutics North America Inc., Montreal, QC H4S 1Z9, Canada; amancini@domaintherapeutics.com (A.M.); samya.aouad@umontreal.ca (S.A.); herthana.kandasamy@mail.mcgill.ca (H.K.); sanmoris@hotmail.com (S.M.); 3 Faculté de Pharmacie, Université Claude Bernard-Lyon, 69373 Lyon, France; 4 Department of Biochemistry and Molecular Medicine, Institute for Research in Immunology and Cancer (IRIC), Université de Montréal, Montreal, QC H3T 1J4, Canada; arhamat@yahoo.fr (A.M.); michel.bouvier@umontreal.ca (M.B.)

**Keywords:** aGPCRs, ADGRE5, BRET, biased signaling, mechanical stimulation, CD55

## Abstract

The adhesion G protein-coupled receptor ADGRE5/CD97 is upregulated in many cancers, representing a potential drug target in oncology/immuno-oncology. Yet, ADGRE5′s activation and signaling mechanisms remain poorly understood. Here, we used enhanced bystander bioluminescence resonance energy transfer (ebBRET)-based biosensors and three strategies to characterize human (h) ADGRE5 signaling. First, a synthetic tobacco etch virus (TEV) protease-cleavable receptor chimera enabling controlled tethered agonist (TA) exposure at the GPCR proteolysis site (GPS) revealed signaling through Gα12 and Gα13, along with the recruitment of β-Arrestins 1/2 (β-Arrs). Second, we investigated WT hADGRE5 signaling elicited by Gingipain K (Kgp), an endopeptidase that cleaves hADGRE5 upstream of the GAIN domain. Kgp mirrored TEV-induced signaling but also promoted Gαz and Gα11 activity. The abolition of hADGRE5′s GPS did not block Kgp-induced receptor activation, revealing a GPS cleavage-independent mechanism of action. Finally, we developed an assay to study hADGRE5 mechanical stimulation (MS) using β-Arr2 as a readout. MS promoted β-Arr2 recruitment in hADGRE5-expressing cells, and this response was lost upon abolition of the GPS. A neutralizing antibody to the hADGRE5 ligand CD55 significantly dampened MS-induced β-Arr2 engagement. Overall, this study advances our understanding of hADGRE5′s signaling and highlights the receptor’s plasticity in activating pathways via both GPS cleavage-dependent and -independent mechanisms.

## 1. Introduction

The adhesion G protein-coupled receptor (aGPCR) family, consisting of 33 distinct members in humans, has emerged as a novel and important group of signaling proteins involved in various physiological processes such as cellular communication, organ development, immune responses, axonal guidance and cell proliferation [1,2,3,4]. They also play a role in various pathological processes underlying neurological and inflammatory disorders, infectious responses, and cancer [5,6,7,8]. Although they possess the typical GPCR architecture of seven transmembrane domains (7TM) interspersed with extracellular and intracellular loops, one of their hallmarks is the presence of an exceptionally long extracellular domain (ECD) containing modular adhesive folds that mediate cell–cell and cell–extracellular matrix interactions [9,10]. The ECD also contains an important and unique feature of aGPCRs, namely the GPCR autoproteolysis-inducing (GAIN) domain. This domain harbors a GPCR proteolysis site (GPS) enabling catalytic self-cleavage during receptor maturation in the endoplasmic reticulum. This event unmasks a cryptic N-terminal sequence acting as a tethered agonist (TA). The consequence of this autoproteolytic process is the generation of two receptor subunits, the N- and C-terminal fragments (NTF and CTF), that remain non-covalently bound and are transported as one unit to the plasma membrane [11,12,13]. Different mechanisms have been proposed to explain the activation of aGPCRs. The tunable model (or allosteric agonism) involves receptor conformational changes induced by NTF-interacting ligands without complete dissociation of the receptor subunits [14,15,16]. In contrast, the shedding model, also known as the stalk dependent model or orthosteric agonism, involves physical dissociation of the NTF and CTF receptor subunits and subsequent stimulation of the aGPCR with an exposed TA [12,17]. These activation mechanisms are not mutually exclusive; individual aGPCRs may be activated in more than one manner [18,19], with the activation mode being determined by the cellular environment and intermolecular interactions [19].

aGPCRs have recently emerged as key modulators in processes related to cancer, namely proliferation, survival, and metastasis [20]. ADGRE5 (or CD97) was the first aGPCR to be associated with cancer [21]. It is mainly expressed (but not restricted to) immune cells [22] and is upregulated in many types of cancers compared with the corresponding normal tissues [23,24,25]. The NTF of ADGRE5 consists of epidermal growth factor (EGF)-like repeats, followed by an Arg-Gly-Asp (RGD) tripeptide sequence and the GAIN domain. ADGRE5 has been implicated in favoring localized tumor growth, enhancing cellular migration, and promoting metastatic spread across various cancer types [26,27,28,29]. Among the interaction partners proposed for ADGRE5, CD55 stands out as an immune system component that is dysregulated in many cancers alongside ADGRE5 [30]. Studies indicate that ADGRE5 and CD55 are involved in different stages of cancer development [24,25,31,32], highlighting the potential of ADGRE5 as a therapeutic target in oncology/immuno-oncology [33]. Despite its therapeutic potential, developing drugs against ADGRE5 has been complicated by an incomplete understanding of ADGRE5′s pharmacology, activation, and signaling mechanisms.

We present herein a comprehensive characterization of human (h) ADGRE5 isoform 2′s signaling repertoire, following different receptor activation modes. We characterized the receptor’s signaling profile toward G proteins and β-Arrestins (β-Arrs) with enhanced bystander bioluminescence resonance energy transfer (ebBRET)-based biosensors [34], using two proteolytic activation methods: (i) a synthetic tobacco etch virus (TEV)-based receptor cleavage assay and (ii) a physiological *Porphyromonas gingivalis* Gingipain K (Kgp) protease-mediated receptor cleavage approach. We also developed an in cellulo mechanical stimulation (MS) BRET-based assay to study physiological (adhesion-mediated) receptor activation. Our findings reveal that hADGRE5 displays a complex signaling behavior, exhibiting biased signaling and both GPS autoproteolysis-dependent and -independent activation. The data also highlight hADGRE5′s mechanosensing ability, with CD55 playing an important role in hADGRE5’s mechanical activation. Overall, these data highlight the spectrum of mechanisms underlying receptor function and establish assays for discovering ligands that can modulate hADGRE5 activity via orthosteric and allosteric sites.

## 2. Materials and Methods

### 2.1. Cells

The HEK293 clonal cell line (HEK293SL cells), hereafter referred to as HEK293 cells, and β-Arrestins 1/2 knockout HEK293 cells (HEK293 β-Arrs KO cells) were a gift from S. Laporte (McGill University, Montreal, QC, Canada) and previously described [35,36]. Cells were maintained in Dulbecco’s Modified Eagle Medium (DMEM) without sodium pyruvate (Wisent, Saint-Jean-Baptiste, QC, Canada) supplemented with 10% fetal bovine serum (Wisent) and 1% antibiotics (100 U/mL penicillin and 100 μg/mL streptomycin; Wisent). Cells were grown at 37 °C in 5% CO_2_ and 90% humidity and checked for mycoplasma contamination.

### 2.2. Plasmids and Constructs

The human (h) ADGRE5 isoform 2 WT (EGF 1,2,5) (UniProtKB identifier P48960-2) coding sequence was synthesized and cloned into pcDNA3.1(+) (TOP Gene Technologies, Montreal, QC, Canada). The plasmid encoding the hADGRE5 isoform 2 WT-Rluc fusion protein was obtained by PCR amplification of the hADGRE5 isoform 2-coding sequence and subcloned in-frame at the N terminus to the Rluc8 sequence into a pcDNA3.1 vector (linker sequence: GSGGGSGGGAS) (TOP Gene Technologies). The hADGRE5 isoform 2 S438A point mutant was produced using the corresponding WT plasmid (TOP Gene Technologies). Sequences encoding Gα_s_-117-RlucII, GFP10-Gγ_1_ [34,37,38], β-Arrestin1-RlucII [39], β-Arrestin2-RlucII [38], rGFP-CAAX [35], Rap1GAP-RlucII, p63RhoGEF-RlucII, p115RhoGEF-RlucII, and PDZRhoGEF-RlucII [34,40] were previously described. Full-length, untagged Gαi1, Gαi2, Gαi3, Gαz, GαoA, GαoB, Gαq, Gα11, Gα14, Gα15/16, Gα12, Gα13, and Gβ1 were purchased from the cDNA Resource Center. Sequences coding for human GRK5 (UniProtKB identifier P34947), human µ-type opioid receptor (hMOR) (UniProtKB identifier P35372-1), and human Somatostatin receptor type 2A (hSST2AR) (UniProtKB identifier P30874-1) were synthesized and cloned between the BamHI and Xba1 sites of a pcDNA3.1 (+) vector (TOP Gene Technologies). Plasmids encoding human Vasopressin V2 receptor (hV2R) and human thromboxane A2 receptor α (hTPα) were provided by Dr. Michel Bouvier (Université de Montréal, Montreal, QC, Canada). The plasmid for the human δ-opioid receptor (hDOR) was a gift from Dr. Louis Gendron (Université de Sherbrooke, Sherbrooke, QC, Canada). Plasmid encoding the human angiotensin II type 1 receptor (hAT1R) was kindly provided by Dr. Sylvain Meloche (Université de Montréal, Montreal, QC, Canada).

DNA encoding hGLP-1R/hADGRE5-TEV (tobacco etch virus) chimera was synthesized by Integrated DNA Technologies (IDT, Coralville, IA, USA) using the mammalian expression vector pCDH-CMV-Ef1-puro (IDT). This construct was designed exactly as described in [41] with the following two sequence modifications: (i) the enterokinase substrate sequence was replaced with a TEV protease cleavage sequence, and (ii) the ADGRG6 C-terminal fragment sequence was replaced with the sequence coding for the C-terminal fragment of hADGRE5. The final construct contained the following 7 contiguous sections: (1) modified hemagglutinin signal peptide (Influenza A virus; amino acids: MAKTIIALSYIFCLVFAQG; with Kozak sequence 5′-ACCATGGCC-3′), (2) human GLP-1R ectodomain amino acids (28–129; ATVSL…CEES), chosen by analyzing the GLP-1R ectodomain structure (PDBID:3IOL), (3) flexible linker GGSGGSGGS (analysis of the Enterokinase light chain structure (PDB ID:1EKB)), (4) combined enterokinase substrate FLAG-tag sequence DYKDDDDK, (5) TEV sequence ENLYFQ, (6) ADGRE5 C-terminal fragment (i.e., β subunit): Human ADGRE5 N-terminally truncated at the natural GPS cleavage site (equivalent to NCBI reference Isoform 2 ID: NP_001775.2 amino acids 438 to 742: SSFAIL…SGI), (7) 3 × HA-tag with stop codon YPYDVPDYAYPYDVPDYAYPYDVPDYA*.

### 2.3. Proteases and Ligands

The TEV protease plasmid was provided by Dr. Jean-Bernard Denault (Université de Sherbrooke, Sherbrooke, QC, Canada); TEV protease (residues 2038–2279 of the TEV polyprotein) was expressed like caspase-3 [42] and dialyzed in 50 mM Tris-HCl, pH 8.0, 50 mM NaCl, 1 mM EDTA, 5 mM DTT, without glycerol. TEV protease was stored at −80 °C. *Porphyromonas gingivalis* protease gingipain K (Kgp) was kindly provided by Dr. Jan Potempa (University of Louisville, Louisville, KY, USA).

DAMGO and Deltrophin are from the peptide synthesis platform of Université de Sherbrooke (Sherbrooke, QC, Canada); Vasopressin (AVP) Peptide, U466619, Angiotensin II (AngII), and SST-14 were purchased from Anaspec (Cat. No AS-24289; Fremont, CA, USA), bio-techne Tocris (Cat. No. 1932; Toronto, ON, Canada), Phoenix Pharmaceuticals (Cat. No. CEK002-12; Burlingame, CA, USA), and Bachem (Cat. No. 4033009; Torrance, CA, USA), respectively.

### 2.4. Transfection and Cell Plating

Transfections were performed as described [38]. Briefly, a polyethyleneimine (PEI) solution (1 mg/mL D-PBS 1X; pH 7.0; Polysciences, Warrington, PA, USA) was mixed (3 μg PEI:1 μg DNA) with biosensor-encoding DNA and the following amounts of receptor-encoding DNA: 250 ng of hADGRE5 WT, 500 ng of hGLP-1R/hADGRE5-TEV, or 25 ng of Rluc-tagged hADGRE5. β-Arrestin1 and 2 biosensors were supplemented with a plasmid encoding human GRK5. All conditions were adjusted to equal final DNA concentrations per mL of cell suspension with salmon sperm-DNA (Invitrogen, Burlington, ON, Canada). The DNA–PEI mixture was incubated at room temperature (RT) for 30 min.

For BRET and internalization assays, cells at a density of 3.5 × 10^5^ were added to the DNA–PEI transfection mix and immediately seeded (3.5 × 10^4^ cells/well) into 96-well white microplates (Greiner Bio-one; Monroe, NC, USA). For the MS-BRET assay, cells at a density of 1.5 × 10^6^ cells/mL were added to the DNA–PEI mixture and directly seeded in 75 cm^2^ T-flasks (1.5 × 10^7^ transfected cells/flask). In parallel, for mechanical stimulation experiments, non-transfected HEK293 cells were seeded in 96-well white microplates at 3.5 × 10^4^ cells/well. Cells were maintained as described in the cell culture section for 48 h until the day of the BRET measurement. For cAMP measurements, the DNA–PEI mixture was added drop by drop to cells previously seeded in 6-well plates (1.5 × 10^5^ cells/well). Cells were kept in culture for 48 h until they were processed for the experiments.

### 2.5. Bioluminescence Resonance Energy Transfer (BRET) Measurement and Mechanical Stimulation (MS) Assay

For BRET assays with TEV protease: at 48 h post-transfection, the cells were washed and incubated in TEV buffer (150 mM Tris-HCL, 0.5 mM EDTA, 1 mM DTT, pH 7.5) for 1 h at 30 °C. A final concentration of 2.5 µM of the luciferase substrate e-Coelenterazine Prolume Purple (Methoxy e-CTZ, Cat. No. 3692; NanoLight Technology, Pinetop, AZ, USA) was added 5 min before reading the BRET. A 3-cycle baseline reading was performed before adding TEV protease (1.25 µM final concentration), and continuous readings were then taken at 30 °C approximately every minute for 40 min.

For BRET assays with Kgp protease: at 48 h post-transfection, cells were washed and incubated in BRET buffer (10 mM Hepes, 1 mM CaCl_2_, 0.5 mM MgCl_2_, 4.2 mM KCl, 146 mM NaCl, 5.5 mM glucose, pH 7.4) for 1 h at 37 °C. A final concentration of 2.5 µM of the luciferase substrate e-Coelenterazine Prolume Purple (Methoxy e-CTZ; NanoLight Technology) was added 5 min before reading the BRET. An initial endpoint reading was recorded to establish the baseline and then increasing concentrations of Kgp were added to the cells. Endpoint readings were taken at 37 °C every 5 min for 1 h.

The readings were recorded on a Berthold TriStar2 LB 942 Multimode Reader (Berthold, Bad Wildbad, Germany) equipped with high-sensitivity BRET^2^ filters (RLucII emission 410 nm/GFP10 emission 515 nm).

For MS-BRET assays: non-transfected cells seeded into 96-well plates for 48 h were washed and incubated in Hanks’ Balanced Salt Solution (HBSS; Wisent) for 1 h at room temperature. Cells transfected in a 75 cm^2^ T-flask were dissociated using Accutase (Corning, Tewksbury, MA, USA), resuspended in DMEM, counted, centrifuged (1200 RPM for 3 min), and resuspended in HBSS to 4 × 10^6^ cells/mL. For the CD55 neutralization assay: transfected cells were incubated with either an anti-CD55 neutralizing antibody (5 µg/mL; monoclonal Mouse IgG2B Clone # 278803, R&D systems, Minneapolis, MN, USA) or an isotype control (5 µg/mL; Monoclonal Mouse IgG2B Clone # 133303, R&D systems) for 1 h at RT in suspension with agitation (250 RPM on Titramax 1000 shaker; Heidolph-instruments, Wood Dale, IL, USA).

At the time of BRET measurement, 10 µL of 20 µM e-Coelenterazine Prolume Purple (Methoxy e-CTZ; NanoLight Technology) was added to plates coated with non-transfected cells followed by the addition of transfected cells (2 × 10^5^/well). Plates were incubated for 5 min at RT, and the BRET was then measured (t = 0 read) on both plates using the Synergy Neo microplate reader (Agilent Technologies, Mississauga, ON, Canada). Increasing doses of the ligand for the control receptor were then added with the D300e Digital Dispenser (Tecan, Morrisville, NC, USA) in corresponding wells. At this point, one of the two plates was subjected to MS (i.e., orbital shaking at 1350 RPM on Titramax 1000 shaker; Heidolph-instruments), and the BRET signals from both plates were recorded after approximately 1, 3, 5, 7, 10, 20, 30, 40, 50, and 60 min of MS.

BRET signals were determined by calculating the ratio of the light intensity emitted by the acceptor (GFP10 or rGFP) over the light intensity emitted by the donor (RlucII). Curve fitting and statistical analyses were performed using GraphPad Prism software. (version 10).

### 2.6. cAMP Measurement

For the measurement of cAMP, the cAMP Gs Dynamic kit (Cat. No. 62AM4PEB; Revvity, Waltham, MA, USA) was used. Cells were transfected as described previously with 250 ng of hADGRE5 WT. The final DNA amount was adjusted to 1000 ng using an empty vector (pcDNA). At 48 h post-transfection, the cells were detached, quantified, and transferred to a 384-well white plate (5000 cells/well). Cells were processed following the manufacturer’s instructions. Cells were incubated overnight under gentle agitation (200 rpm) before being read using a Tecan infinite M1000 Multimode Reader (Tecan, Morrisville, NC, USA) controlled by i-Control™ software (version 1.10).

The donor (620 nm) and acceptor (665 nm) fluorescence were recorded, and the homogeneous time-resolved fluorescence (HTRF) ratio was calculated (665 nm/620 nm). The concentration of cAMP was calculated using the equation derived from the standard curve values.

### 2.7. Flow Cytometry

Cells were transfected with hADGRE5 WT or hADGRE5 S438A in 75 cm^2^ T-flask as described previously. Cells were harvested as described for the MS-BRET assay and were then resuspended in ice-cold PBS/FBS 5% at a density of 10 × 10^6^ cells/mL; 1 × 10^6^ of those cells were incubated for 30 min at 4 °C in the dark with monoclonal anti-hADGRE5 (clone VIM3b) antibody conjugated with FITC (1:10 dilution; BD Biosciences, Mississauga, ON, Canada) or anti-CD55 antibody (IA10) conjugated with APC (1:5 dilution; BD Biosciences) or their respective isotype control (FITC Mouse IgG1 κ 1:10 dilution, APC mouse IgG2a 1:5 dilution; BD Biosciences). Cells were washed 2 times (5 min centrifugation at 1500 RPM at 4 °C), resuspended in PBS/FBS 5% and events were recorded on a LSR II flow cytometer (BD Biosciences). Flow cytometry analysis was performed using FlowJo software (version 10.8.1) (BD Biosciences).

### 2.8. Data Analysis

Data are expressed as means ± standard error of the mean (SEM) of at least three independent experiments. Statistical analysis was performed with GraphPad Prism software (version 10). The type of statistical test and *p* values are indicated in figure legends.

## 3. Results

### 3.1. TA-Dependent Activation of hADGRE5 Reveals Selective G Protein Signaling and β-Arrestin Recruitment

**Figure 1:**
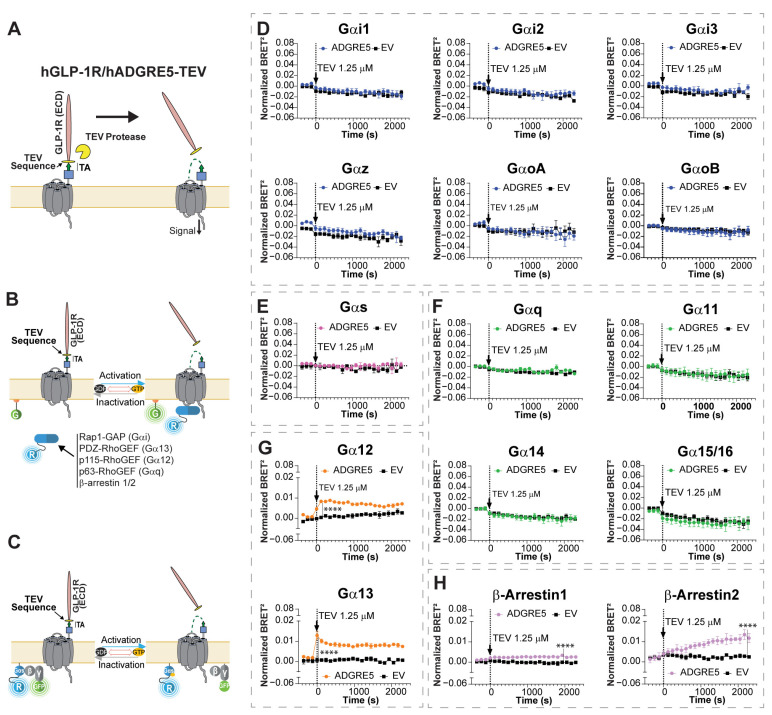
Exposure of hADGRE5′s tethered agonist enhances G protein-dependent activity through Gα12/13 and promotes the engagement of β-Arrestins. (**A**) Schematic representation of the topology and activation mechanism of the synthetic hGLP-1R/hADGRE5-TEV chimera. For the design of the chimera, the N-terminal fragment of hADGRE5 was replaced by the ectodomain of the human glucagon-like peptide 1 receptor (hGLP-1R), and the GPS was replaced with a TEV protease recognition sequence, keeping the tethered agonist (TA) intact (green arrow). Following treatment with TEV protease, the N-terminal fragment of the chimera is released, exposing hADGRE5′s TA and subsequently inducing receptor activation. (**B**) Illustration depicting the G protein Effector Membrane Translocation Assay (GEMTA) to monitor Gα protein activation. Upon receptor activation, RlucII (R)-tagged effector proteins (in blue) interact with active Gα subunits from each G protein family, bringing the Effector-RlucII protein into close physical proximity with plasma membrane (PM)-anchored rGFP (G; in green), ultimately increasing BRET; created with BioRender.com. (**C**) Illustration of the BRET-based conformational Gαs sensor. Upon GPCR-mediated Gαs activation, the RlucII-tagged Gαs-Gβ/γ_1_-GFP10 complex undergoes conformational rearrangement, resulting in the physical distancing between Gαs-RlucII (in blue) and Gγ_1_-GFP10 (in green) and leading to a decreased BRET signal; created with BioRender.com. (**D**–**H**) Time course of the activation of different Gα protein families (Gαi (**D**), Gαs (**E**), Gαq (**F**), Gα12/13 (**G**)) and β-Arrs (**H**), following TEV-induced cleavage of cells expressing hGLP-1R/hADGRE5-TEV chimera (black arrow). Data are represented as the mean values ±  SEM of 3 independent experiments. Normalization of the data was performed by subtracting the results of vehicle treatment from the cells expressing hADGRE5 or empty vector (EV). *p* values were calculated using two-way ANOVA with Šídák’s multiple comparisons test and describe the significance between the point that showed the maximum response in cells expressing hADGRE5 and its counterpart in control cells (EV). **** *p* < 0.0001.

To explore the signaling potential of hADGRE5, we first conducted a broad multi-pathway analysis using a synthetic receptor chimera construct. This approach enables controlled receptor cleavage at the GPS and subsequent exposure of the TA, facilitating real-time monitoring of receptor signaling immediately following TA-mediated activation. For the design of the synthetic chimeric receptor (hGLP-1R/hADGRE5-TEV), we replaced the NTF of hADGRE5 with the ectodomain of hGLP-1 to promote expression and stabilize the folded receptor [41]. The GPS was replaced with a tobacco etch virus (TEV) protease recognition sequence. The treatment of hGLP-1R/hADGRE5-TEV chimera-expressing cells with the TEV protease results in the release of the NTF, leaving the TA exposed and accessible to activate the receptor (Figure 1A).

To assess receptor signaling, we co-expressed the chimera with enhanced bystander BRET (ebBRET) biosensors targeting various G protein pathways. These sensors, collectively known as the “G protein Effector Membrane Translocation Assay (GEMTA)”, use RlucII-tagged G protein effector protein subdomains (p63-RhoGEF, Rap1GAP, p115-RhoGEF, and PDZ-RhoGEF) that translocate to the plasma membrane upon activation of Gq/11, Gi/o/z, G12, and G13, respectively, increasing the ebBRET signal with a membrane-anchored rGFP (Figure 1B) [34]. This membrane translocation strategy is also used to measure β-Arrs engagement [34,35]. For Gαs, we used a RlucII-tagged Gαs and a GFP10-tagged Gγ1 [43]. Receptor activation causes their dissociation, resulting in a decreased BRET signal (Figure 1C) [37,38,44].

We first validated the functionality of the ebBRET-based sensors using well-characterized GPCRs and their specific ligands (Figure S1). We then treated hGLP-1R/hADGRE5-TEV chimera-expressing cells with 1.25 µM of TEV protease. No change in BRET signals was observed for the Gαi/o family G proteins, Gαs, nor Gαq/11 G proteins following TEV protease treatment (Figure 1D–F). In contrast, an increase in the BRET signals indicating coupling to Gα12 and Gα13 was observed (Figure 1G). Of note was that TEV protease had no effect on cells not expressing the hGLP-1R/hADGRE5-TEV chimera (empty vector (EV) controls) (Figure 1, black lines).

In addition to G protein-mediated signaling, GPCRs engage β-Arrs [45]. β-Arrs not only mediate desensitization and endocytosis but also serve as scaffolds to activate various kinase pathways [46,47]. It was reported that hADGRE5 (isoform 3) induces β-Arr2 recruitment in patient-derived glioblastoma cells [33]. We therefore employed ebBRET-based biosensors (Figure 1B) [35] to evaluate whether NTF release and subsequent TA exposure could recruit β-Arr1 and 2. As shown in Figure 1H, treatment of hGLP-1R/hADGRE5-TEV chimera-expressing cells with TEV protease led to the recruitment of both β-Arr1 and 2. Interestingly, β-Arrs recruitment exhibited slower kinetics compared with Gα12 and Gα13 activation (Figure 1G) and was slower than β-Arrs recruitment by non-adhesion GPCRs (e.g., hAT1R in Figure S1E).

### 3.2. Kgp-Mediated Cleavage Highlights hADGRE5 Signaling Complexity Through Induction of a GPS Cleavage-Independent Activation Mechanism

**Figure 2:**
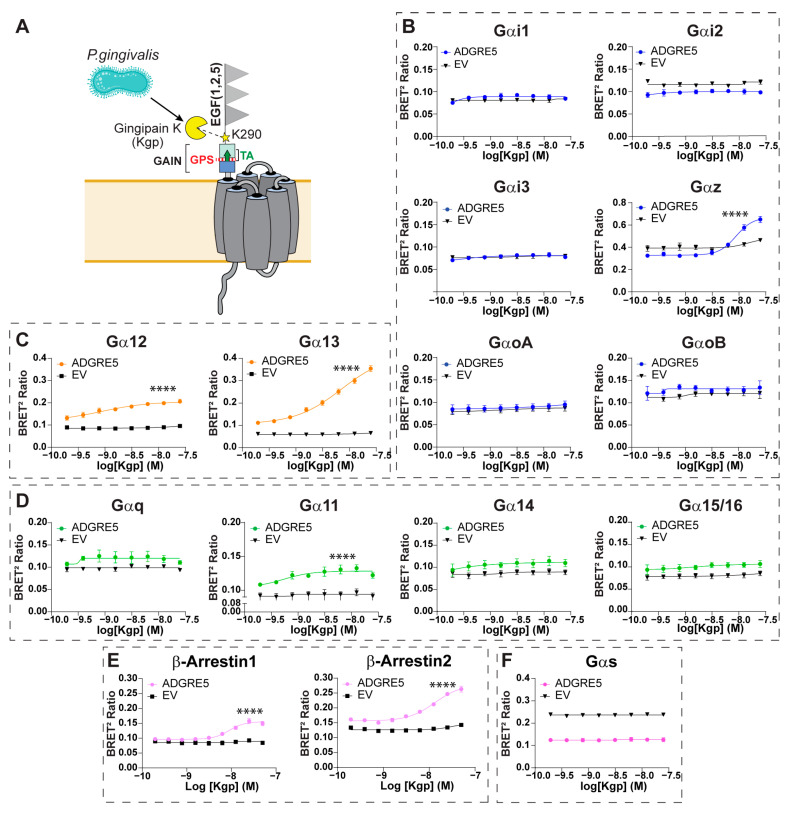
Gingipain K-induced proteolysis of hADGRE5 generates a distinct signaling profile relative to cleavage at the GPS. (**A**) Schematic representation of the topology of hADGRE5, highlighting the site (K290; yellow star) at which Gingipain K (Kgp) cleaves the receptor. (**B**–**F**) Concentration response curves after Kgp-induced activation of different Gαi/o family G proteins (**B**), Gα12/13 (**C**), Gαq family G proteins (**D**), β-Arrs (**E**), and Gαs (**F**). G protein responses were measured 10 min after Kgp addition, while β-Arr data was collected 30 min after Kgp addition. In (**B**–**E**), an increase in the signal denotes pathway activation; in contrast, activation of Gαs (**F**) is denoted by a decreased BRET signal. As a negative control (black line), cells transfected with an empty vector (EV) were subjected to the same conditions as hADGRE5-expressing cells. Data are represented as the mean values ±  SEM of 3 independent experiments. Kgp dose–response curves for EV- and hADGRE5-transfected cells were generated using the log (agonist) vs. response—variable slope (four parameters) non-linear regression model. Curves were compared statistically using the Extra Sum-of-Squares F test, which determines if the best-fit values of various parameters differ between the hADGRE5 and EV curves. **** *p* < 0.0001.

The protease Gingipain K (Kgp) from the oral pathobiont *Porphyromonas gingivalis* was recently shown to cleave hADGRE5 WT at K290 and induce receptor activation [48]. This site lies within the NTF, upstream of the GAIN domain and the GPS (S438). The signaling profile resulting from cleavage at this non-canonical site remains unknown. We therefore used the ebBRET-based sensors (Figure 1B,C) to assess the signaling response following the Kgp-induced cleavage of hADGRE5 WT (Figure 2A).

Treatment of hADGRE5-expressing cells with increasing concentrations of Kgp resulted in a dose-dependent activation of Gαz, Gα12, Gα13, Gα11, and β-Arrs (Figure 2B middle right; Figure 2C; Figure 2D second graph; Figure 2E). Pathway activation kinetics were also evaluated. Unlike the rapid, transient G protein activation (Figure S2A–C), β-Arrs recruitment began ~1000 s after Kgp treatment and increased steadily thereafter (Figure S2D). No response occurred in cells lacking hADGRE5.

Notably, hADGRE5-expressing cells exhibited constitutive activation of Gαs, which was unaffected by Kgp-induced cleavage (Figure 2F). To validate this observation, we measured cAMP levels, the second messenger of the Gαs pathway. Consistently, hADGRE5-expressing cells showed significantly higher cAMP levels compared with control cells transfected with an empty vector (Figure S3).

**Figure 3:**
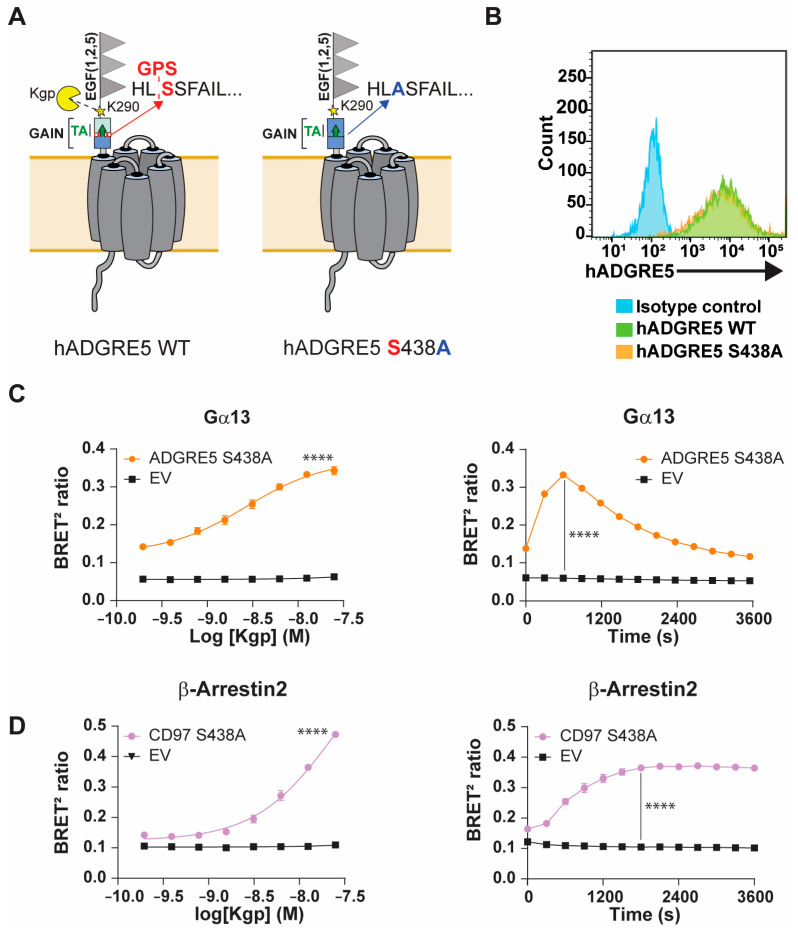
The autoproteolysis-deficient hADGRE5 mutant preserves its ability to signal and recruit β-Arrestins. (**A**) Schematic representation of the topology of hADGRE5 WT (left) and the autoproteolytic cleavage-deficient hADGRE5 S438A mutant (right). (**B**) Flow cytometric analysis of cell surface hADGRE5 WT (green) and GPS-mutant (S438A) (orange) expression. (**C**,**D**) Concentration response curves (left) and time course (right) of the activation of Gα13 (**C**) and β-Arr2 engagement (**D**) following Kgp-induced cleavage of hADGRE5 S438A. Data are represented as the mean values ±  SEM of 4 independent experiments. Kgp dose–response curves for EV- and hADGRE5-transfected cells were generated using the log (agonist) vs. response—variable slope (four parameters) non-linear regression model. Curves were compared statistically using the Extra Sum-of-Squares F test, which determines if best-fit values of various parameters differ between the hADGRE5 and EV curves. *p* values for the time course were calculated using two-way ANOVA with Šídák’s multiple comparisons test and describe the significance between the point that showed the maximum response in cells expressing hADGRE5 S438A and its counterpart in control cells (EV). **** *p* < 0.0001.

The GAIN domain of aGPCRs and exposure of the TA following NTF dissociation and/or rearrangement are thought to drive receptor activation [19]. Because GPS autoproteolysis enables movement within the GAIN domain, disrupting this cleavage could clarify how Kgp mediates hADGRE5 activation. We therefore generated a hADGRE5 S438A mutant, replacing the GPS P1′serine with alanine to block GPS autocleavage (Figure 3A) [49]. We first compared the cell surface expression of hADGRE5 WT and the autocleavage-deficient mutant by flow cytometry and found both constructs were expressed at identical levels (Figure 3B). We next examined the pathways most robustly activated by Kgp cleavage of hADGRE5 WT, namely Gα13 and β-Arr2. In hADGRE5 S438A-expressing cells, Kgp induced a dose-dependent activation of both pathways (Figure 3C,D, left). Interestingly, whereas the Gα13 response was comparable to WT (Figure 3C left vs. Figure 2C right), β-Arr2 recruitment was enhanced in the autocleavage-deficient mutant (Figure 3D left vs. Figure 2E right). The Gα13 activation kinetics matched those observed with the WT receptor (Figure 3C right vs. Figure S2C right), but β-Arr2 recruitment occurred faster in the S438A mutant, reaching WT maximal levels earlier (Figure 3D right vs. Figure S2D right).

Overall, our findings demonstrate that hADGRE5 has a diverse signaling repertoire, with site-specific NTF cleavage causing signaling bias. Cleavage with TEV activates Gα12/13 and promotes the recruitment of β-Arr1 and 2 through TA exposure. In contrast, Kgp cleavage engages Gα12/13 and β-Arrs but also activates Gαz and Gα11 via a GPS cleavage-independent mechanism. Moreover, real-time kinetics experiments reveal distinct temporal activation patterns of effectors, underscoring the receptor’s complex pharmacology.

### 3.3. Human ADGRE5 Is Activated by Mechanical Stimulation

**Figure 4:**
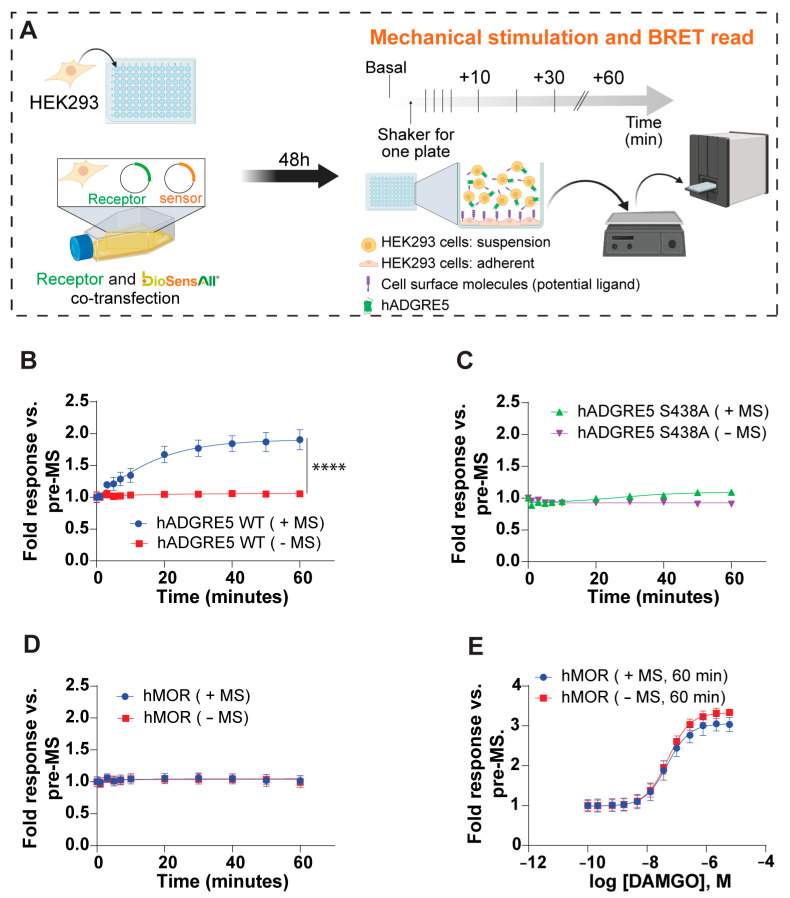
Mechanical stimulation (MS) of human ADGRE5 induces β-Arrestin2 recruitment to the plasma membrane. (**A**) Diagram illustrating the general workflow for the MS assay. HEK293 cells were co-transfected with hADGRE5 and the β-Arr2 sensor. After 48 h, the co-transfected cells were lifted and added to a 96-well plate that was pre-coated with parental non-transfected HEK293 cells. The plate was subjected to MS via vigorous shaking on a standard orbital shaker and the BRET signal was recorded at various time points; created with BioRender.com. (**B**) β-Arr2 plasma membrane (PM) recruitment after subjecting (blue) or not subjecting (red) hADGRE5 WT-expressing cells to MS over a period of 60 min. (**C**) Impact of mutating the hADGRE5 GPS (S438A) on MS-induced β-Arr2 recruitment to the PM. (**D**) Control experiment assessing β-Arr2 PM recruitment after subjecting (blue) or not subjecting (red) hMOR-expressing cells to MS for 60 min. (**E**) Concentration response curves of agonist (DAMGO)-induced β-Arr2 PM engagement in hMOR-expressing cells subjected (blue) or not (red) to MS for 60 min. β-Arr2 PM recruitment data are represented as the mean values ±  SEM of 3–4 independent experiments. *p* values were calculated using two-way ANOVA with Šídák’s multiple comparisons test and describe the significance between the point that showed the maximum response in cells expressing hADGRE5 subjected to MS (+MS) and control cells not subjected MS (−MS). **** *p* < 0.0001.

In vivo, aGPCRs are believed to serve as mechanosensors that convert extracellular mechanical cues into intracellular signaling [50,51,52,53]. To better understand if and how hADGRE5 is activated through mechanical stimulation (MS) [54], we developed an in cellulo BRET-based assay (MS-BRET) where cells co-transfected with hADGRE5 and the β-Arr2 engagement biosensor were subjected to vigorous MS on an orbital shaker (Figure 4A). The β-Arr2 engagement biosensor was selected as a readout since it generated a robust and persistent signal with the previous activation strategies. Figure 4B shows that cells transfected with hADGRE5 and subjected to MS exhibit a robust and sustained recruitment of β-Arr2 to the PM. No change in BRET was observed in the absence of MS. To test whether GPS autoproteolysis contributes to MS-induced activation, we applied the MS-BRET assay to cells expressing the S438A mutant. The mutation abolished β-Arr2 recruitment following MS (Figure 4C), indicating that GPS cleavage (and possibly TA exposure) is required for MS-driven signaling.

In addition, the MS of cells expressing a control GPCR, the human μ opioid receptor (hMOR), did not evoke a response (Figure 4D) despite hMOR’s ability to recruit β-Arr2 upon agonist (DAMGO) stimulation (Figure 4E). These data demonstrate that the observed effect is a specific cellular response mediated by hADGRE5 and not a broad effect of MS on the β-Arr2 biosensor.

### 3.4. Kgp-Mediated Cleavage and Mechanical Stimulation Induce hADGRE5 Internalization

**Figure 5:**
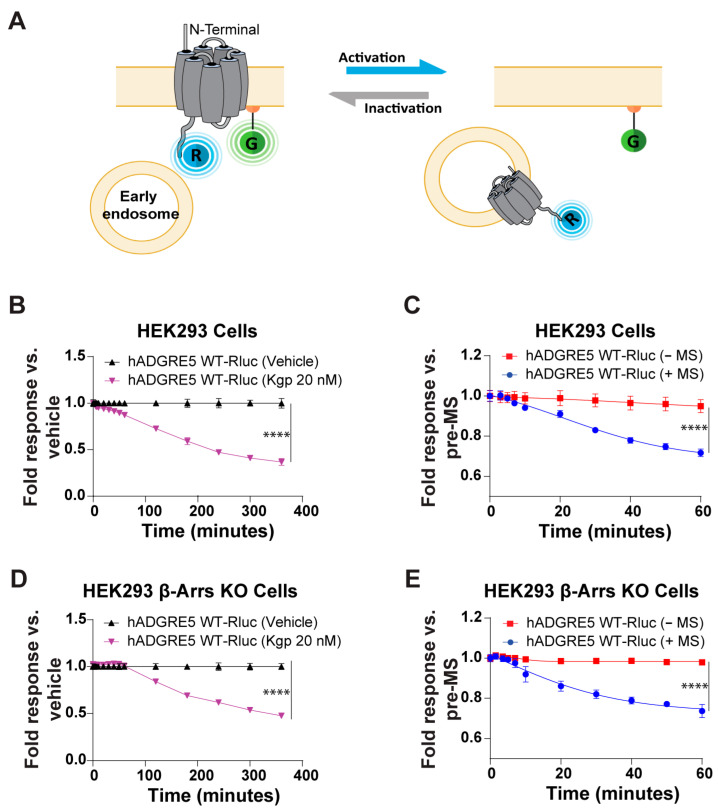
Kgp-induced stimulation and mechanical stimulation (MS) lead to human ADGRE5 internalization. (**A**) Illustration of the receptor trafficking sensor, which consists of the receptor fused at its C-terminus to RlucII (R; in blue) and membrane-anchored rGFP (G; in green). Internalization of the tagged receptor results in a time-dependent decrease in the BRET signal; created with BioRender.com. (**B**) Evaluation of hADGRE5 WT-RlucII internalization after Kgp-induced stimulation (purple) or vehicle (black) over a period of 400 min. (**C**) Evaluation of hADGRE5 WT-RlucII internalization after subjecting (blue) or not subjecting (red) receptor-transfected cells to MS over a period of 60 min. (**D**,**E**) Same as in (**B**,**C**) but using receptor-transfected HEK293 β-Arr 1 and 2 knockout cells. Data are represented as the mean values ±  SEM of 2–4 independent experiments. *p* values were calculated using two-way ANOVA with Šídák’s multiple comparisons test and describe the significance between the point that showed the maximum response in cells expressing hADGRE5 after Kgp-induced cleavage and control (vehicle) in (**B**,**D**), or cells expressing hADGRE5 subjected to MS (+MS) and control cells not subjected MS (−MS) in (**C**,**E**). **** *p* < 0.0001.

GPCR activation is frequently followed by receptor endocytosis, a process mediated by various cellular mechanisms. Among these, β-Arrs are the most well-characterized mediators of receptor internalization [55,56]. To evaluate whether Kgp- or MS-induced activation of hADGRE5 leads to receptor internalization, we used a BRET receptor trafficking assay comprising a Rluc-tagged version of hADGRE5 and a PM-anchored rGFP. Receptor internalization produces a decrease in the BRET signal (Figure 5A). As shown Figure 5B, Kgp-induced hADGRE5 cleavage led to a significant reduction in BRET relative to vehicle-treated cells. Similarly, receptor internalization was detected in hADGRE5-expressing cells subjected to MS, while no significant reduction in BRET signal was observed in cells not subjected to MS (Figure 5C). To further validate the MS-induced internalization of hADGRE5, we repeated the assay with a Rluc-tagged version of the somatostatin hSST2A receptor. The hSST2A receptor is not known to respond to MS but internalizes following binding to somatostatin. Indeed, no change in BRET was seen with MS, but hSST2A-Rluc internalized upon stimulation with somatostatin-14 (SST-14) (Figure S4).

Finally, we sought to determine whether β-Arrs recruitment is mechanistically linked to hADGRE5 internalization. To address this, we assessed receptor internalization in HEK293 β-Arrs KO cells [36] following Kgp- or MS-induced activation. As shown in Figure 5D,E, hADGRE5 continues to be internalized in response to both stimuli even in the absence of β-Arrs. However, in the β-Arrs KO cells, hADGRE5 internalization following Kgp-mediated cleavage was delayed, beginning at ~100 min (vs 40 min in parental HEK293 cells) (Figure 5D vs. Figure 5B). Collectively, these results reveal that hADGRE5 undergoes β-Arrs-independent internalization following Kgp- and MS-induced activation.

### 3.5. The ADGRE5 Ligand CD55 Is Required for Mechanical Stimulation-Induced β-Arrestin2 Recruitment

**Figure 6:**
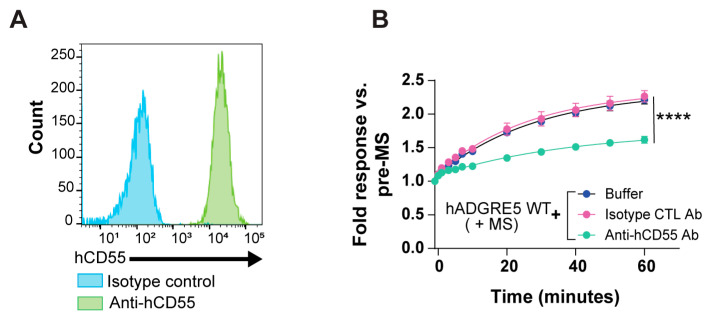
Mechanical stimulation (MS)-induced β-Arrestin2 engagement by human ADGRE5 involves receptor interaction with CD55. (**A**) Flow cytometric analysis of cell surface CD55 expression in parental HEK293 cells used in this study. (**B**) Analysis of cell surface CD55 blockade on MS-induced hADGRE5 β-Arr2 recruitment to the plasma membrane (PM). hADGRE5-transfected HEK293 cells were incubated with a neutralizing anti-hCD55 antibody (teal), an isotype control (CTL) antibody (pink), or assay buffer alone (blue). Cells were then subjected to MS, and the β-Arr2 response recorded over a period of 60 min. β-Arr2 PM recruitment data are represented as the mean values ±  SEM of 3 independent experiments. *p* values were calculated using two-way ANOVA with Šídák’s multiple comparisons test and describe the significance between the point that showed the maximum response in cells expressing hADGRE5 treated with the anti-hCD55 antibody and cells expressing hADGRE5 treated with an isotype control antibody. **** *p* < 0.0001.

Studies have demonstrated that ADGRE5 and its ligand CD55 are co-expressed in cells of several tumor types. This interaction has been associated with tumor progression and increased aggressiveness [57,58,59,60,61]. Importantly, the hADGRE5 isoform 2-hCD55 complex was shown to display mechanical force-resisting shearing stretch geometry, potentially enabling a mechanosensing mechanism for hADGRE5 activation and signaling [62]. We thus investigated whether the interaction of hADGRE5 with CD55 is implicated in mediating β-Arr2 recruitment in our MS-BRET assay. First, we used flow cytometry to confirm that CD55 is expressed on the surface of our HEK293 cells (Figure 6A). We then assessed MS-mediated β-Arr2 recruitment in hADGRE5 expressing cells in the presence of a neutralizing antibody that prevents CD55`s interaction with hADGRE5. Cells were also treated with an isotype control antibody alone as a specificity control of the CD55 neutralizing antibody. Our results showed a significant reduction in the BRET signal in the presence of the CD55 neutralizing antibody compared with control conditions (Figure 6B), indicating that CD55 participates in the MS-mediated recruitment of β-Arr2 to hADGRE5.

## 4. Discussion

hADGRE5′s multifaceted roles in cancer have been well documented. This receptor has been shown to inhibit apoptosis, regulate tumor cell survival, and modulate various tumorigenic mechanisms, including tumor cell adhesion, migration, and invasion in various cancers [25,61,63,64,65]. In addition to its role in tumor cells, ADGRE5 is also postulated to have a role in immuno-oncology. Indeed, predictive models centered on hADGRE5 expression in T cells within the tumor microenvironment have been developed to identify responders to neoadjuvant cancer immunotherapy [66]. These findings highlight ADGRE5′s potential as a therapeutic target. A deeper understanding of its signaling could clarify its functions in cancer progression and immune responses and support the development of new targeted therapies. We performed an in-depth characterization of hADGRE5′s signaling signature using various ebBRET-based sensors and three distinct activation methods to better understand the biology and pharmacology of the receptor.

Recent high-resolution structures of hADGRE5 in both active and inactive states have shed light on the mechanisms that govern receptor activation [67]. While prior studies have highlighted that ADGRE5 preferentially couples to the Gα13 pathway [29,67,68,69], our proteolysis-based activation experiments further demonstrated coupling to Gα12 and the recruitment of β-Arr1 and 2. Interestingly, the cleavage of hADGRE5 by Kgp, but not TEV protease, resulted in the activation of Gαz and Gα11. It is worth emphasizing that while the TEV protease-induced activation of hADGRE5 uses a synthetic receptor construct where TEV protease cleaves at the GPS to expose the TA, Kgp-induced activation uses the natural receptor with cleavage occurring at a different site than the GPS, namely residue K290. This difference in NTF proteolytic cleavage sites suggests that Kgp may activate hADGRE5 through a mechanism distinct from TEV, particularly with regard to the role and contribution of the TA. Using the hADGRE5 S438A mutant, we show that blocking GPS autoproteolysis does not impair Kgp-induced activation. These results are consistent with previous reports revealing that non-cleavable aGPCRs retain their signaling capability [70,71]. Indeed, spontaneous TA exposure has been reported in five intact aGPCR homologs, including hADGRE5 [13]. Moreover, several aGPCRs have been shown to function through TA-independent mechanisms, both in vitro and in vivo [14,15,72,73]. Although the precise contribution of the TA to Kgp-mediated activation requires further investigation, our data indicate that Kgp activity does not require NTF dissociation or full TA exposure.

Although our data suggest a signaling bias (i.e., hADGRE5 can couple to different pathways based on the activation mode), we cannot fully discount the possibility that technical aspects unique to the TEV and Kgp assays may account for these observations. Indeed, this signaling bias elicited by differential hADGRE5 cleavage could be partly explained by TEV’s slower catalytic rate (*k_cat_* of 0.18 s^−1^) [74,75] compared with Kgp’s catalytic rate (up to 10 s^−1^) [76]. Receptor activation by Kgp may thus provide greater sensitivity in detecting weaker hADGRE5 G protein couplings (i.e., Gα11 and Gαz) relative to the TEV protease approach. Similarly, the detection of β-Arr1 recruitment using TEV-induced activation is very limited, whereas it became more robust when using Kgp-induced activation.

Additionally, we identified constitutive activity of hADGRE5 on Gαs, which we validated by measuring cAMP production. Given that this stimulus-independent response was not observed with the synthetic TEV-cleavable version of the receptor (which lacks a complete GAIN domain) (Figure 1), we deduce that autoproteolytic processing at the GPS and intra-GAIN domain movements or isomerization of the TA are important for the constitutive activation of the Gαs pathway. The biological relevance and mechanism of coupling to pathways regulating cAMP production (i.e., Gαs and Gαi/o/z) remains to be determined. Interestingly, the cAMP-PKA axis is emerging as an important checkpoint in the immune response, and its dysregulation has been linked to various cancers [77,78]. Altogether, these data provide the most comprehensive hADGRE5 signaling map to date, unmasking a possible signaling bias dependent on the proteolytic activation method used.

While engineered aGPCRs with modified or absent NTFs serve as valuable tools for identifying potential signaling effectors [79,80], these synthetic approaches have limitations in fully mimicking the physiological activation mechanisms of native receptors. Such artificial constructs, while informative, may not capture the nuanced regulation and complex interactions that occur in vivo. First, the presence of the NTF is essential to evaluating the effect that interactions with different ligands have on receptor activation and signaling. Second, the use of NTF-deficient aGPCRs precludes the identification of therapeutics acting on this long and tractable region of the receptor. Using the MS-BRET assays described herein, we activated hADGRE5 in cellulo via a mechanism that could mimic certain in vivo activation modalities, thus circumventing the above-described limitations of synthetic approaches. We showed that MS promotes hADGRE5 activation, as highlighted by the engagement of β-Arr2, and receptor internalization. Using a receptor harboring a mutated GPS, we provide evidence that an intact GPS is required for β-Arr2 recruitment following MS. These data may indicate that the increased GAIN domain flexibility conferred by GPS autoproteolytic cleavage is important for hADGRE5 mechanotransduction but not for Kgp-mediated signaling.

Generally, there are at least two requirements for aGPCR activation in vivo: (i) the anchoring of the aGPCR NTF to a ligand present on adjacent cells or in the extracellular matrix (ECM), combined with (ii) movement of the NTF relative to the anchored ligand. This movement generates sufficient force to induce a conformational rearrangement and/or dissociation of the NTF, leading to TA unmasking and subsequent signaling [81]. The MS-BRET assays described here incorporate both elements, with intercellular hADGRE5-CD55 interactions combined with MS promoting hADGRE5 mechanotransduction. Of the four known ADGRE5-ligand interactions, CD55 is the best characterized. In cancer, ADGRE5 and CD55 are co-expressed and upregulated in several tumors, with higher expression often correlated to increased disease severity and poor prognosis [58,59,60,82]. The hADGRE5-CD55 interaction has also been clearly shown to regulate various immune functions, including immune synapse stabilization and ADGRE5 downregulation on circulating leukocytes [83,84]. Recently, Liu et al. [68] highlighted the importance of the ADGRE5-CD55 interaction for splenic dendritic cell (DC) localization and homeostasis in vivo. Specifically, contact between ADGRE5-expressing type-2 conventional DCs (cDC2s) and circulating CD55-expressing red blood cells (i.e., condition of shear stress) was shown to promote the extraction of ADGRE5′s NTF, leading to TA exposure and activation of the Gα13 pathway. This pathway subsequently promotes the anchoring of cDC2 cells at the tissue–blood interface, optimizing antigen capture and allowing for efficient immune responses to systemic pathogens. The MS-BRET-based assays preserve the hADGRE5-CD55 interaction and mimic such mechanical stress conditions. We also demonstrated the participation of the hADGRE5-CD55 complex in MS-induced β-Arr2 recruitment. Similarly, the interaction of ADGRE5 with CD90 was shown to lead to the recruitment of β-Arr2, subsequently activating the MAPK signaling pathway [33]. The ability of hADGRE5 to recruit β-Arrs and to undergo internalization upon activation prompted us to investigate whether these processes are mechanistically linked. Experiments using β-Arrs KO HEK293 cells demonstrated that hADGRE5 internalized via β-Arrs-independent mechanisms, thereby adding this receptor to the expanding list of GPCRs capable of endocytosis through alternative pathways [85,86]. The functional consequences of β-Arrs engagement by hADGRE5 remain to be elucidated but may involve roles in signaling—such as MAPK pathway activation—and/or receptor internalization-independent desensitization and signal termination.

In addition to mimicking physiological activation on a pathophysiologically relevant signaling pathway, the MS-BRET assays may have a potential utility in drug screening applications. Indeed, use of the full-length receptor would permit the identification of drugs targeting the long NTF with at least three modes of action/modalities that are distinct from binders acting at the orthosteric site: (1) allosteric modification of receptor conformation, leading to altered TA unmasking; (2) interference with NTF–ligand interaction (as exemplified by the neutralizing anti-CD55 antibody data in Figure 5B); and (3) anti-NTF targeting antibodies that are conjugated to drugs (as was shown in [33] for glioblastoma) or have cell-depleting activities (e.g., antibody-dependent cellular cytotoxicity, antibody-dependent cellular phagocytosis, or complement-dependent cytotoxicity).

In summary, this study provides new insights into hADGRE5 signaling using two proteolytic activation methods, revealing the activation of Gα12, Gα13, Gαz, and Gα11 and the recruitment of β-Arr1 and 2. We introduce MS-BRET assays to study receptor activation in cellulo under physiological conditions and identify CD55′s role in β-Arr2 recruitment after MS. Importantly, distinct activation modes show different GPS autoproteolysis requirements, highlighting hADGRE5′s adaptability to diverse stimuli. Collectively, the described methodologies and protocols are applicable to other aGPCRs and mechanosensitive GPCRs.

## Data Availability

All data and analysis are included in the main text of the manuscript and the supporting material. All BRET-based biosensors described are freely available under the material transfer agreement for academic research from the Université de Montréal and can be requested from MB.

## Supplementary Materials

The following supporting information can be downloaded at: https://www.mdpi.com/article/10.3390/cells14161284/s1, Figure S1: Control experiments confirming the functionality of BRET-based biosensors; Figure S2: Signaling pathways triggered by Kgp cleavage of hADGRE5 exhibit distinct activation kinetics; Figure S3: hADGRE5 expression increases basal cAMP production; Figure S4: Internalization of the control receptor after mechanical stimulation and stimulation with its natural ligand.
