# Supplementary material for: Decoding ADGRE5: How Proteolytic Cleavage and Mechanical Forces Unleash Cellular Signals
Source: Cells. 2025 Aug 19;14(16):1284. doi: 10.3390/cells14161284 (PMC12384904; doi:10.3390/cells14161284)
Supplement: Supplementary file 1 [file cells-14-01284-s001.zip › cells-3795148-supplementary.pdf]

Supplementary figures

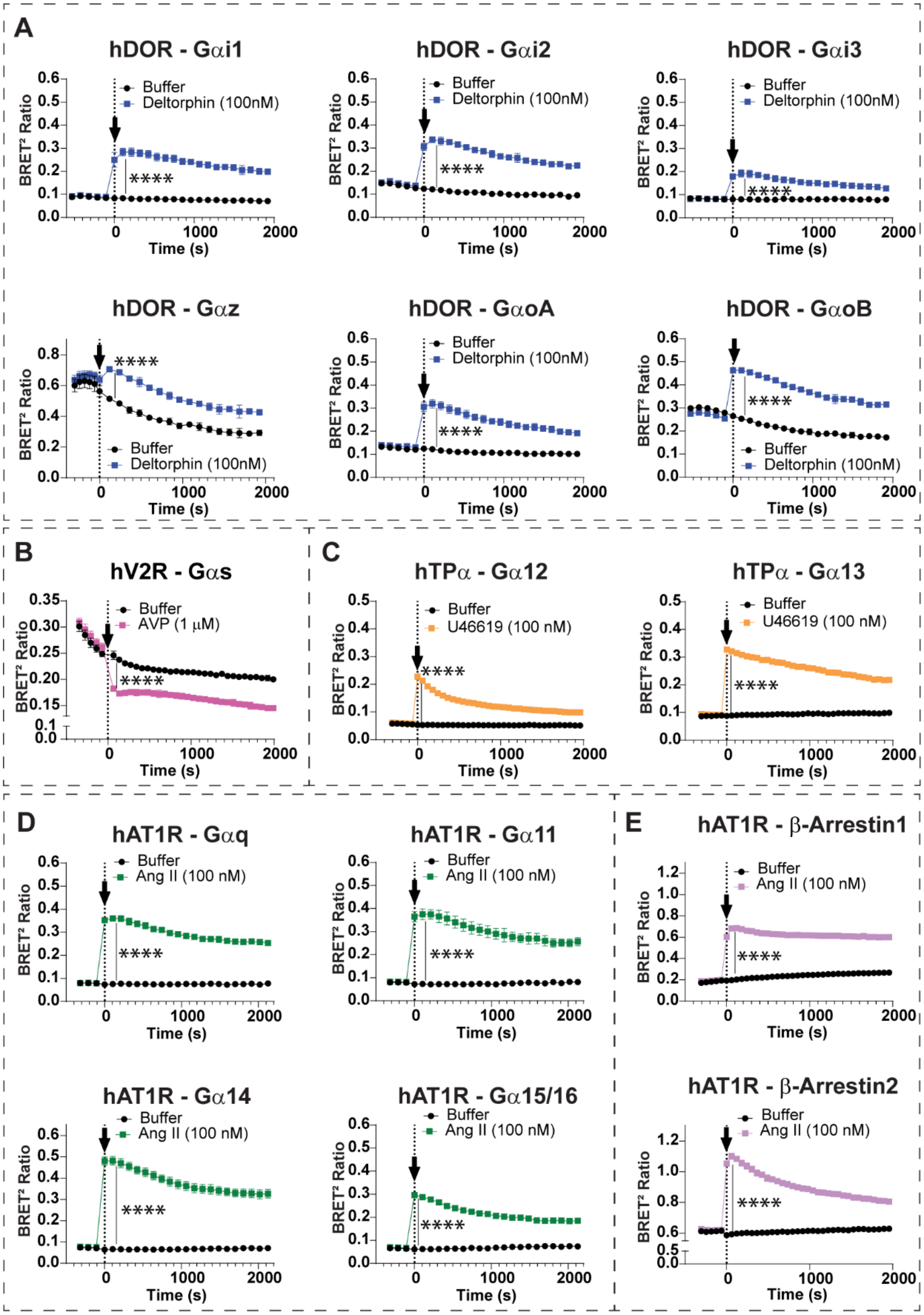

Figure S1. Control experiments confirming the functionality of BRET-based biosensors**.** The functionality of the BRET-based biosensors was confirmed using various well-characterized GPCRs: human δ-opioid receptor (hMOR) stimulated with Deltorphin (100 nM) for Gαi family (**A**), human Vasopressin receptor 2 (hV2R) stimulated with arginine vasopressin (AVP; 1µM) for Gαs (**B**), human thromboxane A2 receptor α isoform (hTPα) stimulated with U46619 (100 nM) for Gα12/13 (**C**), human angiotensin II type 1 receptor (hAT1R) stimulated with angiotensin II (100 nM) for Gαq family (**D**) and β-Arrs (**E**). Black lines represent non-stimulated controls. BRET was recorded approximately every 60 s before and after ligand addition (black arrow). Data are represented as the mean values ± SEM of 3 independent experiments. *p* values were calculated using two-way ANOVA with Šídák's multiple comparisons test and describe the significance between the point that showed the maximum response in stimulated and non-stimulated (Buffer) conditions. **** $p < 0.0001$.

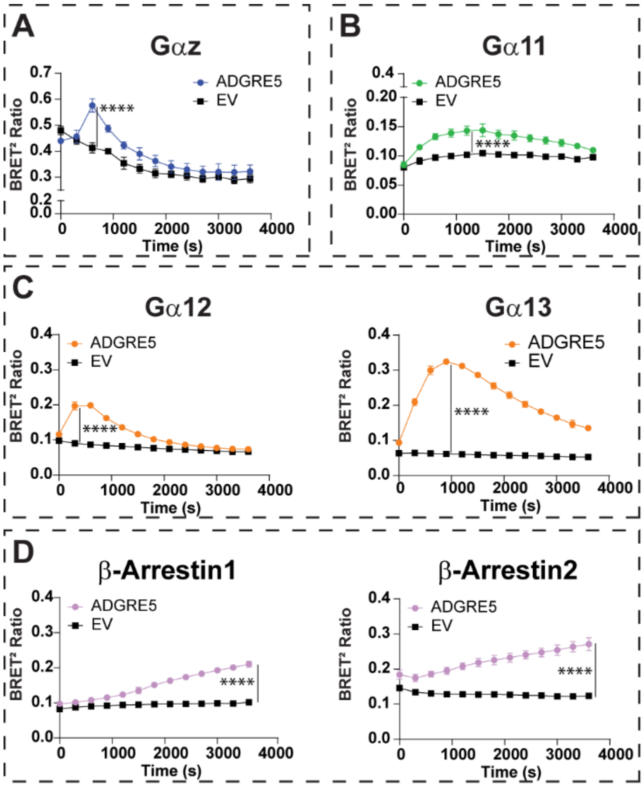

Figure S2. Signaling pathways triggered by Kgp cleavage of hADGRE5 exhibit distinct activation kinetics. Time course of the activation of different Gα proteins (Gαz (**A**), Gα11 (**B**), Gα12/13 (**C**)) and β-Arrs (**D**) following 12.5 nM of Kgp-induced cleavage of hADGRE5. As a negative control (black line), cells transfected with empty vector (EV) were subjected to the same conditions than hADGRE5-expressing cells. BRET was recorded every 300 s following the addition of Kgp. Data are represented as the mean values ± SEM of 3 independent experiments. *p* values were calculated using two-way ANOVA with Šídák's multiple comparisons test and describe the significance between the point that showed the maximum response in cells expressing hADGRE5 and its counterpart in control cells (EV).**** $p < 0.0001$.

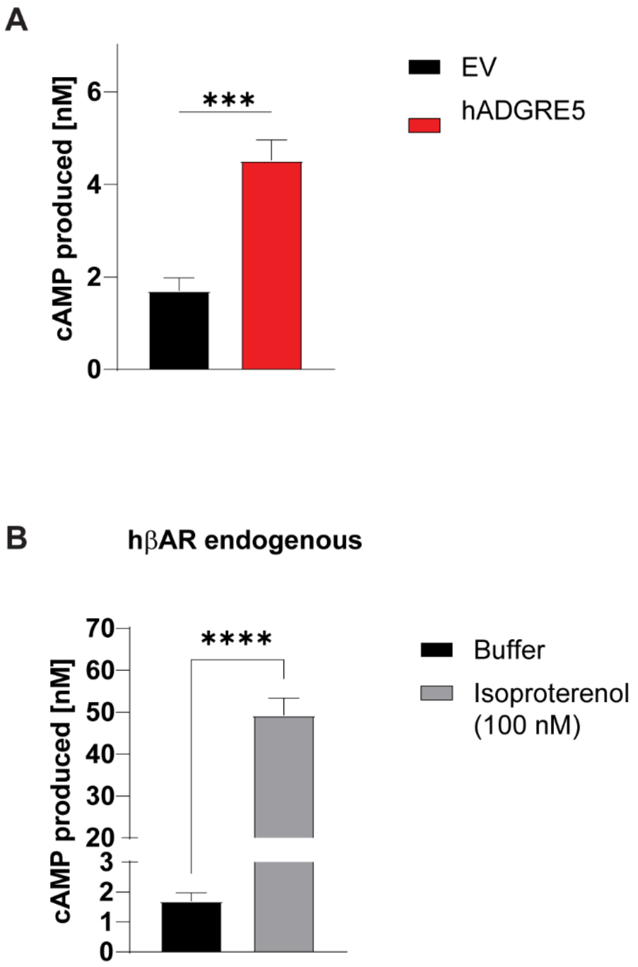

Figure S3. hADGRE5 expression increases basal cAMP production. (**A**) Evaluation of cAMP production in HEK293 cells transfected with hADGRE5. HEK293 cells transfected with empty vector (EV) were used as a reference for basal cAMP levels present in the cells (black). (**B**) cAMP assay control in which HEK293 cells were treated with 100 nM of isoproterenol, leading to stimulation of endogenously-expressed human β adrenergic receptors (hβAR). Data are represented as the mean values ± SEM of 3 independent experiments. p values were calculated using two-way ANOVA with Dunnett's multiple comparisons test and describe the significance between the comparisons indicated in the graphs. *** $p < 0.001$, **** $p < 0.0001$.

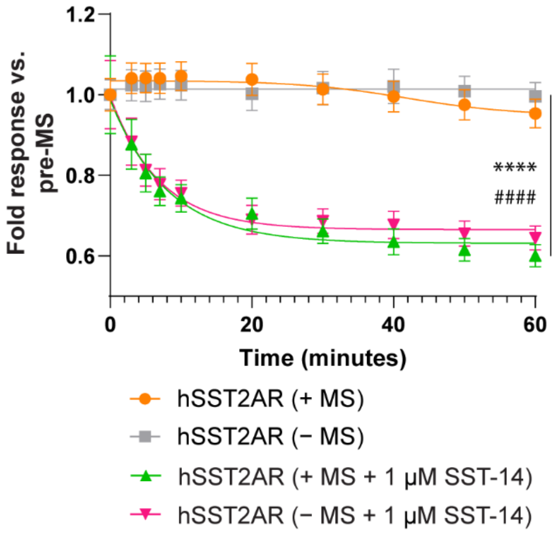

Figure S4. Internalization of the control receptor after mechanical stimulation and stimulation with its natural ligand. Control experiment evaluating hSST2AR-Rluc-internalization following MS (orange, grey) or in response to stimulation with its natural ligand SST-14 (green, pink). Data are represented as the mean values ± SEM of 3 independent experiments. *p* values were calculated using two-way ANOVA with Šídák's multiple comparisons test and describe the significance between the point that showed the maximum response in cells expressing hADGRE5 subjected to MS (+MS) and cells expressing hADGRE5 subjected to MS + 1µM SST-14 (**** $p < 0.0001$), or cells expressing hADGRE5 not subjected to MS (−MS) and cells expressing hADGRE5 not subjected to MS + 1µM SST-14 (#### $p < 0.0001$).
